# Geographical and seasonal distribution of the Short-crested Coquette hummingbird: a microendemic and endangered species

**DOI:** 10.7717/peerj.20312

**Published:** 2025-11-11

**Authors:** Pablo Sierra-Morales, Octavio R. Rojas-Soto, Luis A. Sánchez-González, Carina Gutiérrez-Flores, R. Carlos Almazán-Núñez

**Affiliations:** 1 Doctorado en Recursos Naturales y Ecología, Facultad de Ecología Marina, Universidad Autónoma de Guerrero, Acapulco, Guerrero, Mexico; 2 Red de Biología Evolutiva, Laboratorio de Bioclimatología, Instituto de Ecología, A.C., Xalapa, Veracruz, Mexico; 3 Laboratorio de Procesos Evolutivos y Diversidad de Aves Neotropicales, Facultad de Ciencias, Museo de Zoología “Alfonso L. Herrera”, Departamento de Biología Evolutiva, Universidad Nacional Autónoma de México, Ciudad de México, Mexico; 4 SECIHTI, Escuela Superior en Desarrollo Sustentable, Universidad Autónoma de Guerrero, Tecpan de Galeana, Guerrero, Mexico; 5 Laboratorio de Ecología y Biogeografía de la Conservación, Facultad de Ciencias Químico Biológicas, Universidad Autónoma de Guerrero, Chilpancingo, Guerrero, Mexico

**Keywords:** Hummingbirds, Seasonal climatic niche, Restricted-range species, Elevational distribution, Conservation

## Abstract

Species movements along elevational or latitudinal gradients occur primarily due to climatic variations and food resource availability. However, the role of seasonal climatic conditions in species with highly restricted distributions has been poorly addressed. In this study, we analyzed the geographic distribution and seasonal climatic niche during the dry and rainy seasons of the Short-crested Coquette hummingbird (SCCH; *Lophornis brachylophus*), a species with high conservation priority at the global scale. We generated ecological niche and species distribution models for both seasons and used niche similarity tests to represent and compare their climatic differences. We recorded the availability of flowering and fruiting plants that the SCCH feeds on within its distribution area during both seasons and performed a kernel density analysis to evaluate the main peaks in food availability. Our results revealed that the potential distribution of the SCCH is larger (642 pixels) in the dry season than in the rainy season (487 pixels). In the dry season, the distribution of this hummingbird includes sites at lower elevations (reaching 780 m above sea level [masl]). In contrast, in the rainy season, it extends to higher elevations (up to 1,450 masl). This seasonal shift between the two seasons coincides with the availability of flowers and fruits along the elevational gradient. The climatic niche similarity between the dry and rainy seasons shows moderate overlap (Schoener’s D = 0.50) and is higher than expected by chance. Our results suggest that the SCCH moderate changes in its climatic niche throughout the year, with plant phenology being a primary driver of changes in its elevational range between seasons.

## Introduction

Some animal groups, such as birds, undertake latitudinal and elevational movements primarily in response to temporal and spatial variations in food availability and climatic conditions within their distribution range (Dingle & Alistair-Drake, 2007; Jahn et al., 2020; Rojas-Soto et al., 2021). These seasonal or temporal movements depend on dispersal capacity and the physiological tolerance that species have to environmental conditions (Dingle & Alistair-Drake, 2007; Rojas-Soto et al., 2021). In birds, the influence of seasonal climatic conditions, particularly regarding survival or range shifts, has been studied mainly in the context of long-distance migrations (Peña-Peniche, Ruvalcaba-Ortega & Rojas-Soto, 2018; Gutiérrez-Illán et al., 2022; Hernández-Hernández et al., 2022). Conversely, the role of seasonal climatic conditions in bird species engaging in local or elevational movements has received less attention in the scientific literature (Prajzlerová et al., 2025). Moreover, movements of bird species engaging in mutualistic interactions, such as seed dispersers and pollinators, such as hummingbirds, are primarily driven by seasonal climatic variations and/or the availability of food resources, which influence both the species’ movement patterns and distributional range (Abrahamczyk et al., 2011; Hernández-Hernández et al., 2022; Ochoa-González et al., 2023). Although information on hummingbirds’ elevational movements remains limited for many regions, at least 119 of all extant hummingbird species are known to perform such movements (Barçante, Vale & Alves, 2017), mainly due to the limitation or variation of food resources and biotic interactions (Rueda-Uribe et al., 2024).

The paucity of research on microendemic species is mainly attributable to the intrinsic characteristics of the spatially restricted and commonly mountainous environments in which they have evolved (Cordier et al., 2019; Platania & Gómez-Zurita, 2023; Araujo, Machado & da Silva, 2024). In addition, since seasonal climatic conditions along an elevational gradient can show contrasting changes, factors such as temperature, precipitation, and humidity may impose a strong environmental filter on hummingbird distribution (Abrahamczyk et al., 2011), especially for endemic or microendemic species (Graham et al., 2009). Assessments of changes in the geographic distribution of species with restricted ranges and their suitable areas have been driven mainly by the dynamics of climate change and recent distributional shifts, including assessments of their vulnerability and conservation (Cordier et al., 2019; Sierra-Morales et al., 2021; Salazar-Miranda et al., 2024).

Studies analyzing and comparing seasonal climatic niches of microendemic species in geographic and environmental spaces using correlative models remain scarce (Cordier et al., 2019; Rojas-Soto et al., 2021; Toro-Cardona, Parra & Rojas-Soto, 2023; Rivadeneira et al., 2025), particularly in birds. Since microendemic species are more vulnerable to extinction, ecological niche and species distribution models represent a powerful tool to drive effective strategies for their conservation (Giachello et al., 2025). These models can be used to identify key environmental characteristics influencing the probability of a species’ occurrence and, therefore, predict how habitat suitability varies across the landscape (Elith & Leathwick, 2009). Such ecological and distributional information enhances knowledge of physiological responses and constraints to seasonal climate variations (Encarnación-Luévano, Peterson & Rojas-Soto, 2021), as well as the adaptive strategies required for conserving their ecological niches (Rojas-Soto et al., 2021). However, since species with narrow ranges often display habitat specificity at both local and regional scales, they are typically expected to be constrained by climatic conditions (Ohlemüller et al., 2008). Therefore, understanding how microendemic species respond to seasonal climate variations could offer valuable insights into potential adaptations to future climate change, thereby informing more effective conservation strategies.

**Figure 1:**
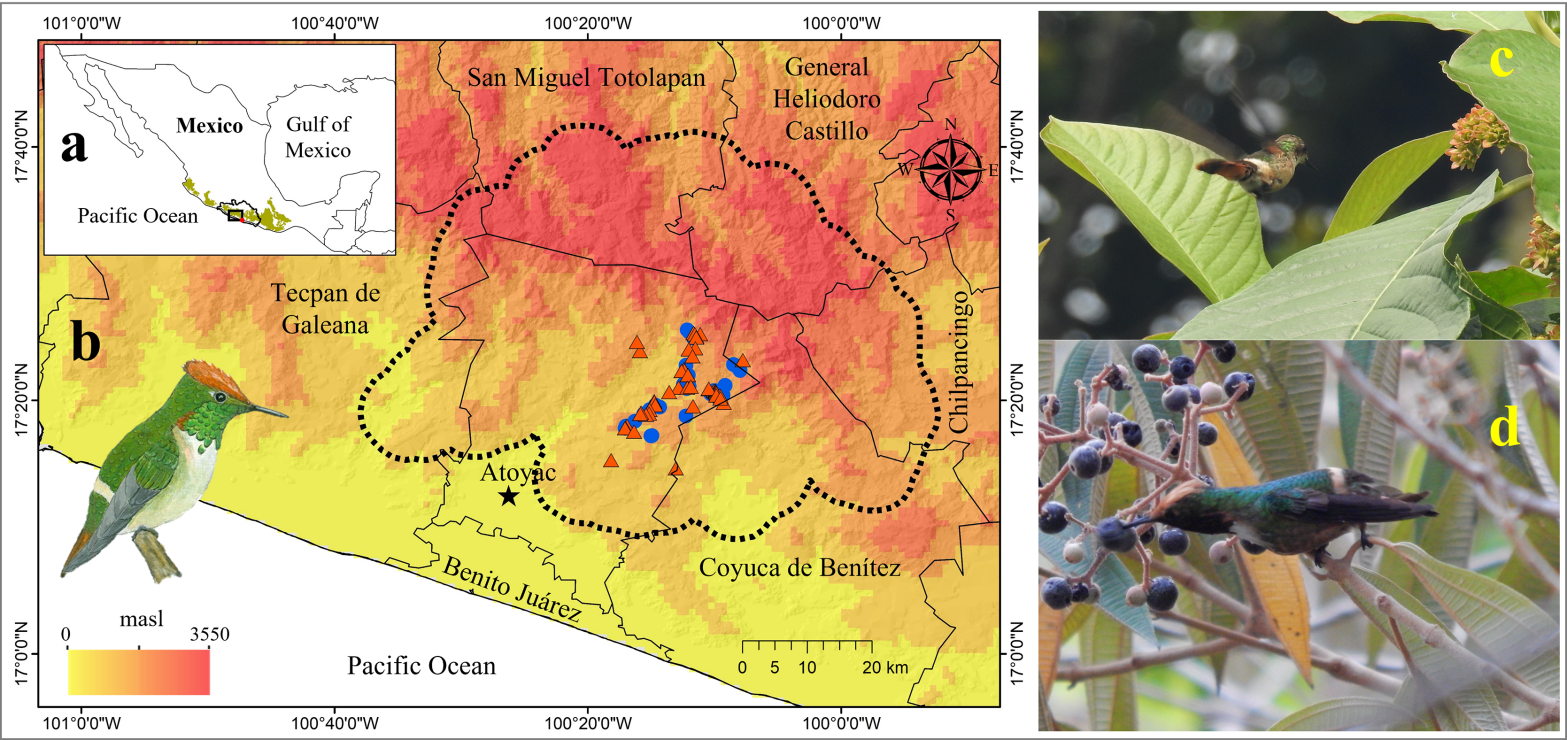
Geographic location of the area occupied by the Short-crested Coquette hummingbird (SCCH). (A) The state of Guerrero in southern Mexico (biogeographic province of the Sierra Madre del Sur is illustrated by the green polygon), (B) localities where SCCH is present, (C) sightings of the SCCH (female) feeding on nectar in the flowers of the *Sommera grandis* tree during the dry season, and (D) sightings of the SCCH (male) feeding on nectar from the fruits of *Conostegia xalapensis* during the rainy season. Municipal boundaries are shown, with records of the species’ presence indicated in red for the dry season and in blue for the rainy season. The image of the SCCH (in (B)) was sourced from “*Aves de la Sierra de Atoyac, Guerrero, México*” (Almazán-Núñez et al., 2024).

The Short-crested Coquette hummingbird (SCCH; *Lophornis brachylophus*) is a species primarily restricted to humid montane habitats, such as cloud forest and tropical semideciduous forest, frequently in combination with shade coffee plantations within a small region of the Sierra Madre del Sur, in southern Mexico (Fig. 1; Banks, 1990; Arizmendi & Berlanga, 2014). Its limited distribution, approximately 90 km^2^, its small population size (reportedly fewer than 1,500 individuals, IUCN, 2024), and particularly the isolated region with security issues where it inhabits, are key factors contributing to the scarcity of information regarding the ecology, demographic processes, life history, evolution and conservation of this hummingbird. However, recent studies have documented the potential effects of land use and climate change on this species’ distribution (Sierra-Morales et al., 2016, 2021; Prieto-Torres et al., 2021). Furthermore, several aspects of SCCH’s natural history are unknown, although it has recently been observed to feed on the nectar from at least 13 plant species (Arizmendi et al., 2021; López-Flores et al., 2024; Bravo-Galindo et al., 2025). Interestingly, its diet during the rainy seasons also includes fruits of *Conostegia xalapensis*, a pioneer shrub associated with shade coffee plantations (P Sierra and RC Almazán, 2021, personal observations). Given the markedly seasonal floral phenology and the importance of sugary fruits for this species in the region, the SCCH possibly undertakes elevational movements in search of food resources.

Understanding species movements, particularly in pollinators such as the SCCH, is crucial for elucidating the significance of ecological and environmental factors in driving spatial and temporal movements (Abrahamczyk et al., 2011). Hummingbirds, particularly widespread ones, show seasonal shifts in their movement patterns and distribution in response to variations in floral resources or climatic conditions (Arizmendi, 2001; Hernández-Hernández et al., 2022). These movements are studied mainly in northernmost populations of widespread species, such as Berylline hummingbird, White-eared hummingbird, and Rivoli’s hummingbird (Lara, 2006; López-Segoviano et al., 2018). Conversely, for microendemic species such as the SCCH, the climatic and biotic conditions that drive shifts in their distribution and seasonal niche remain unknown.

This study aimed to analyze the climatic niche and potential distribution of the SCCH across its range during both the dry and rainy seasons. Like many other hummingbird species, the SCCH tends to undertake elevational movements associated with seasonal climate variation in search of food resources (López-Segoviano et al., 2018). Therefore, we expect a more widespread distribution of this hummingbird during the dry season, when food availability (primarily flowers) is greater along the elevational gradient within its geographic range, compared to the rainy season. However, despite climate variation throughout the year, significant differences are not expected in its seasonal climatic niche due to the species’ localized and restricted distribution.

## Materials and Methods

### Distribution records

Presence records of the SCCH were gathered from online biodiversity repositories, including the Global Biodiversity Information Facility (GBIF, 2018, https://www.gbif.org/es/occurrence/search?taxon_key=2477012&occurrence_status=present) and eBird (eBird, 2018, https://ebird.org/species/shccoq). Additionally, we incorporated records obtained from fieldwork conducted during bimonthly observations spanning the dry (November to April) and rainy (May to October) seasons between 2020 and 2023. Observations were made using 8 × 45 binoculars along transects of 2 to 3 km length during the species’ peak activity hours (6:00 to 11:00 and 16:00 to 18:30). These observations took place in environments typically inhabited by the SCCH, including cloud forests, tropical semideciduous forests, and shade coffee plantations across an elevational gradient ranging from 700 to 1,700 m above sea level (masl; Almazán-Núñez et al., 2024). We compiled a database of 261 presence records, with 211 obtained from online repositories, six from scientific literature, and 50 from fieldwork. This database was projected using a Geographic Information System (ArcMap 10.4) both to verify the accuracy of the record locations and to eliminate incorrectly georeferenced ones (*e.g*., records not corresponding to the species’ habitat, such as oak forests or tropical dry forests), as well as duplicates based on location. This cleaning process yielded a final dataset of 83 records: 49 for the dry season and 34 for the rainy season (Supplemental Information). Based on the restricted distribution and the extinction risk faced by the species, we consider these record numbers adequate for generating species distribution models with a good approximation performance.

### Calibration area

We delimited the calibration area for the modelling process based on the accessibility area approach (Barve et al., 2011), which include regions that may have been accessible throughout the evolutionary history of the species (Soberón & Peterson, 2005; Barve et al., 2011). Considering the occurrence records for the local distribution of the SCCH (~70 to 90 km^2^; Birdlife International, 2024; Almazán-Núñez et al., 2024), the accessible area was defined using the hydrological sub-basins in HydroSHEDS (Lehner & Grill, 2013). These sub-basins belong to the hydrological region Atoyac-Costa Grande (RH19A; Ortiz-Partida et al., 2019). We preferred to use hydrological sub-basins instead of other proposed units, such as ecoregions or biogeographic provinces (Barve et al., 2011; Rojas-Soto et al., 2024), as these latter may overestimate accessible area due to the species’ highly restricted distribution. Furthermore, like ecoregions, sub-basins have biological significance in delimiting species’ presence and dispersal capacity, as they may constitute geographic barriers (Rojas-Soto et al., 2024). Finally, as part of the accessible area, we included a 30 km buffer zone to encompass records located within the boundaries of the 12 identified sub-basins. The calibration area includes cloud forest, pine forest, pine-oak forests, tropical semideciduous forests, tropical dry forests, as well as agricultural and livestock areas (INEGI, 2021). The climate is warm-humid, temperate-subhumid, and semi-cold subhumid, with rainfall ranging from 1,000 to 2,500 mm per year and elevations from 400 to 3,550 masl.

### Bioclimatic variables

To generate potential distribution models of the SCCH for both the rainy and dry seasons, we used seven monthly bioclimatic variables available from WorldClim version 2.1 (see Table S1; Fick & Hijmans, 2017), with a spatial resolution of 0.0083° (~1 km^2^). Each variable was delineated using the species’ accessibility area as a calibration area for modelling.

To reduce the correlation among variables, we performed a variance inflation factor (VIF) analysis (Der & Everitt, 2002) using the *usdm* package (Naimi & Araújo, 2016) in R version 4.0 (R Development Core Team, 2024). This analysis includes an iterative process to identify the least collinear variables (VIF < 10; Zuur, Ieno & Smith, 2007), as has been used in other modelling studies (*e.g*., Dormann et al., 2013; Sierra-Morales et al., 2021). From this procedure, five variables were selected to generate the distribution models for both seasons. Variables such as mean temperature and wind speed were excluded due to a high correlation with the remaining variables (Table S1).

We selected the climatic variables from May (bio 5) to October (bio 10) for the rainy season and from November (bio 11) to April (bio 4) for the dry season (Table S1). The maximum and minimum values of each variable were used to represent the environmental conditions influencing species survival. The presence records of the SCCH were grouped according to the corresponding seasons, as were the bioclimatic variables (Table S1). Finally, values for each climatic variable were extracted from the occurrence records of the SCCH in both dry and rainy seasons. To evaluate the variation of the five variables in both seasons, we used the nonparametric Mann-Whitney U test with the *Wilcox.test* function of the *stats* package in R version 4.0 (R Development Core Team, 2024).

### Potential distribution models

To generate the ecological niche and species distribution models (ENM/SDM), we used the MaxEnt algorithm version 3.4.1 (Phillips, Anderson & Schapire, 2006), implemented in the *kuenm* package (Cobos et al., 2019) in R version 4.0 (R Development Core Team, 2024). This package generates candidate models, which are evaluated to produce final models (Cobos et al., 2019).

The *kuenm* package evaluates models based on the following parameters: (1) statistical significance, which assesses the ratio of the area under the curve (AUC) of the receiver operating characteristic (partial ROC), based on independent random subsets of presence data; (2) predictive performance (omission rate ≤ 5%); and (3) model fit and simplicity, in terms of the Akaike information criterion (AICc < 2 units; Warren & Seifert, 2011).

Two sequences of regularization multiplier values were explored, ranging from 0.1 to 1 with 0.1 as the increment and 2 to 6 with one as the increment. Given the low number of records for the species, three combinations of feature classes were made: quadratic (q), linear (l), and linear-quadratic (lq), represented as follows: “q,” “l,” “lq.” These features smooth the responses of the variables and contribute to reducing model complexity (Phillips, Anderson & Schapire, 2006; Cobos et al., 2019).

After obtaining the best ten models for each modelling exercise, the median of these replicates was selected as a more accurate indicator of the highest frequency in the distribution, as opposed to the mean, which is affected by extreme values (Cobos et al., 2019). For all final models, a cut-off threshold at the tenth percentile of training presence was applied to generate binary distribution models (*i.e*., presence-absence; 0–1), such that the 10% of records with the lowest probability values were excluded. This threshold criterion minimizes overpredictions in the output maps (Liu et al., 2013).

### Seasonal climatic niche similarity

Climatic similarity between the dry and rainy seasons was assessed using Schoener’s D metric for the niche similarity test, whose values range from 0 to 1, where zero indicates no overlap and one indicates complete overlap (Warren, Glor & Turelli, 2008; Broennimann et al., 2012). To interpret the results, we considered the classification of Rödder & Engler (2011): null overlap (0–0.2), low overlap (0.2–0.4), moderate overlap (0.4–0.6), high overlap (0.6–0.8), and very high overlap (0.8–1). To perform the niche similarity test, we used the *ecospat.niche.overlap* function of the *ecospat* package (Di Cola et al., 2017) in R version 4.0 (R Development Core Team, 2024). This function calculates the D overlap metrics based on two grids: (1) occurrence density and (2) environmental variables. This index employs a principal component analysis (PCA-env) to reduce the dimensionality of the variables (Broennimann et al., 2012). This approach transforms the multivariate environmental space into a PCA two-dimensional space, revealing the main environmental gradients, *i.e*., the most relevant explanatory variables for the species’ niche.

Kernel density was applied to smooth the number of species’ presence records in the environmental space cells. To represent the environmental space used by the SCCH, we employed the *ecospat.plot-niche* function of the *ecospat* package implemented in R software version 4.0 (R Development Core Team, 2024). Niche models were generated separately by season.

To test the hypothesis of climatic niche equivalency of the SCCH between the dry and rainy seasons, we used the observed Schoener’s D values to compare with expected Schoener’s D values generated from null models. This process involved 1,000 repetitions, and the “greater” alternative was assigned, where the null hypothesis posits that the niches are more similar than expected by chance (Broennimann et al., 2012; Di Cola et al., 2017).

### Food availability

To integrate biotic factors that may explain potential changes in the geographic distribution of the SCCH between the dry and rainy seasons, we recorded flowering trees or shrubs used as food resources. This species has been observed feeding on the flowers of around 13 plant species, including *Inga vera, Sommera grandis, Clethra fragrans, Coffea arabica*, and *Clusia salvinni*, among others (Fig. 1C; Arizmendi et al., 2021; López-Flores et al., 2024). During the rainy season, this species also consumes nectar from some soft fruits by inserting its bill to extract sugary juice (*e.g*., *Conostegia xalapensis*, Fig. 1D). Frugivory by hummingbirds is an opportunistic behavior increasingly documented (Missagia et al., 2025).

We covered a gradient from 700 to 1,700 masl, conducting three linear transects of approximately 2 km in length at every 200 m of elevation. These transects were surveyed bimonthly during 2023, covering both seasons. For each transect, we recorded the number of plants with flowers or fruits from which this hummingbird feed. Using data on the number of flowering or fruiting plants used by this species, a kernel density function was applied to compare the main peaks of food density along the altitudinal gradient between the two seasons.

## Results

### Geographic distribution

Out of the 84 candidate models built, six met the *kuenm* evaluation criteria, from which we obtained the best potential model (*i.e*., these exhibiting low omission values < 0.02, and good predictive performance distribution) for the SCCH for each of the two seasons. According to the partial AUC values, models in both seasons were significantly better than expected by chance and demonstrated lower complexity, as indicated by the Delta AICc (Table 1).

**Table 1:** Parameterization of the best distribution model of the Short-crested Coquette (*Lophornis brachylophus*) in both seasons.

Season	Model	Feature classes	Regularization multiplier	AUC_ratio	Omission rate	AICc	Delta_AICc
Dry	M_2_F_lq_set_01	lq	0.1	1.55	0.02	712.97	0
Rainy	M_0.4_F_lq_set_01	lq	0.1	1.82	0	452.11	0

**Note:**

*Parameters:* dAIC, Akaike information criterion; AUC, area under the partial ROC curve. *Feature class:* linear (l), quadratic (q).

**Figure 2:**
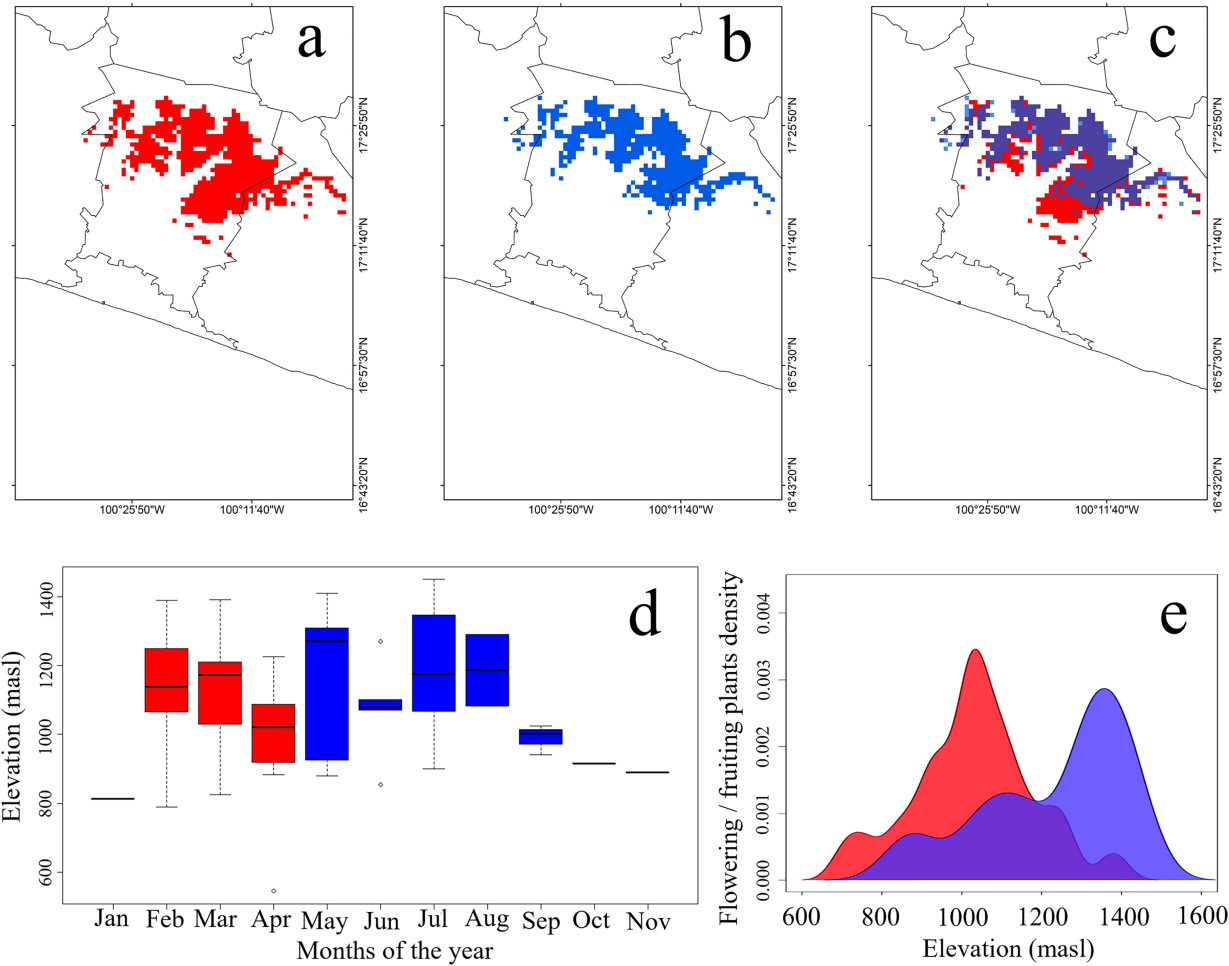
Potential geographic distribution of the Short-crested Coquette hummingbird. (A) During the dry season, (B) during the rainy season, (C) overlap in both seasons, (D) elevational records of presence, and (E) density of flowering plants within the hummingbird’s range. Red indicates the dry season, and blue the rainy season. The purple color in (2E) signifies the overlap of food density between the dry and rainy seasons.

The potential distribution of this species covered 642 pixels in the dry season, and 487 pixels in the rainy season (Figs. 2A–2C). The distribution of the SCCH during the dry season expanded toward the southern portion of the study area, occupying lower elevations between 780 and 800 masl (Fig. 2C and 2D). In this season, 59% of the total predicted area coincides with cloud forest, 32% with tropical semideciduous forest, and 10% with pine-oak forests. In contrast, during the rainy season, this hummingbird increased its elevation range to 1,300 and 1,450 masl (Fig. 2C and 2D). For this season, 66% of its potential distribution overlaps with cloud forest, 27% with tropical semideciduous forest, and 8% with pine-oak forest. Furthermore, the geographical areas where the SCCH can be observed throughout the year overlaps mainly between 900 and 1,300 masl. In the dry season, variables that contributed most to the model were minimum solar radiation, precipitation, and minimum temperature. In contrast, in the rainy season, the most important variables were maximum and minimum solar radiation, as well as the lowest temperature within the range of maximum temperatures (Table S2).

### Food availability and climatic characterization of the SCCH

Plant species used as food resources by the SCCH exhibited two main flowering or fruiting peaks during both the dry and rainy seasons. In the dry season, food resource density reached its peak at elevations close to 750 masl, significantly increasing between 900 and 1,100 masl. In the rainy season, food resource density peaked from 1,100 to 1,450 masl (Fig. 2E). Notably, during this season, *Conostegia xalapensis* is an abundant plant species that provides fruits on which the SCCH feeds (see Fig. 1D). Our observations documented that these fruits are widely consumed by the SCCH, as groups of 4 to 5 individuals foraged on approximately 15 fruits within 20 min, a feeding behavior that had not been previously observed. The highest number of plant species recorded with mature flowers or fruits, these latter of *Conostegia xalapensis*, on which this species feeds occurred at elevations of 1,100 and 1,300 masl (Table S3).

**Figure 3:**
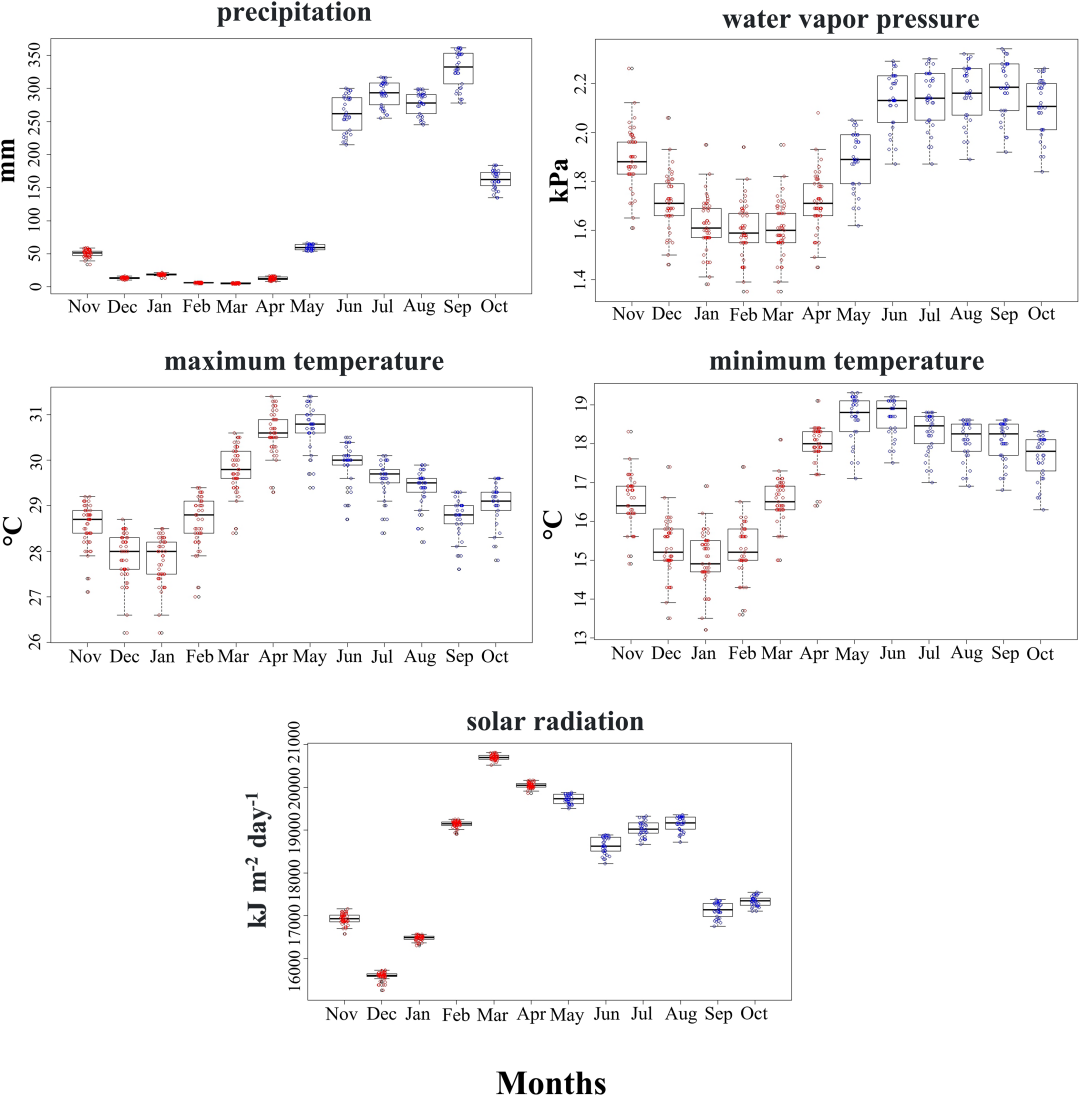
Range of values for the climatic variables used by the Short-crested Coquette hummingbird during the dry (red) and rainy (blue) seasons in its distribution area.

Climatic conditions used by the SCCH in both seasons primarily varied with precipitation, water vapor pressure, and minimum temperature (Mann-Whitney test, *z* = −6.471, *z* = −5.065, *z* = −4.223, *P* < 0.05, respectively; Fig. 3). The other climatic variables, such as maximum temperature and solar radiation, did not differ significantly between the two seasons (Fig. 3). Solar radiation exhibited higher ranges during 2 months of the dry season compared to all the rainy months at the sites used by this species. This variable is particularly important in floral phenology during March and April, marked by the greatest availability of flowers in the area inhabited by this hummingbird.

### Seasonal climatic niche similarity

**Figure 4:**
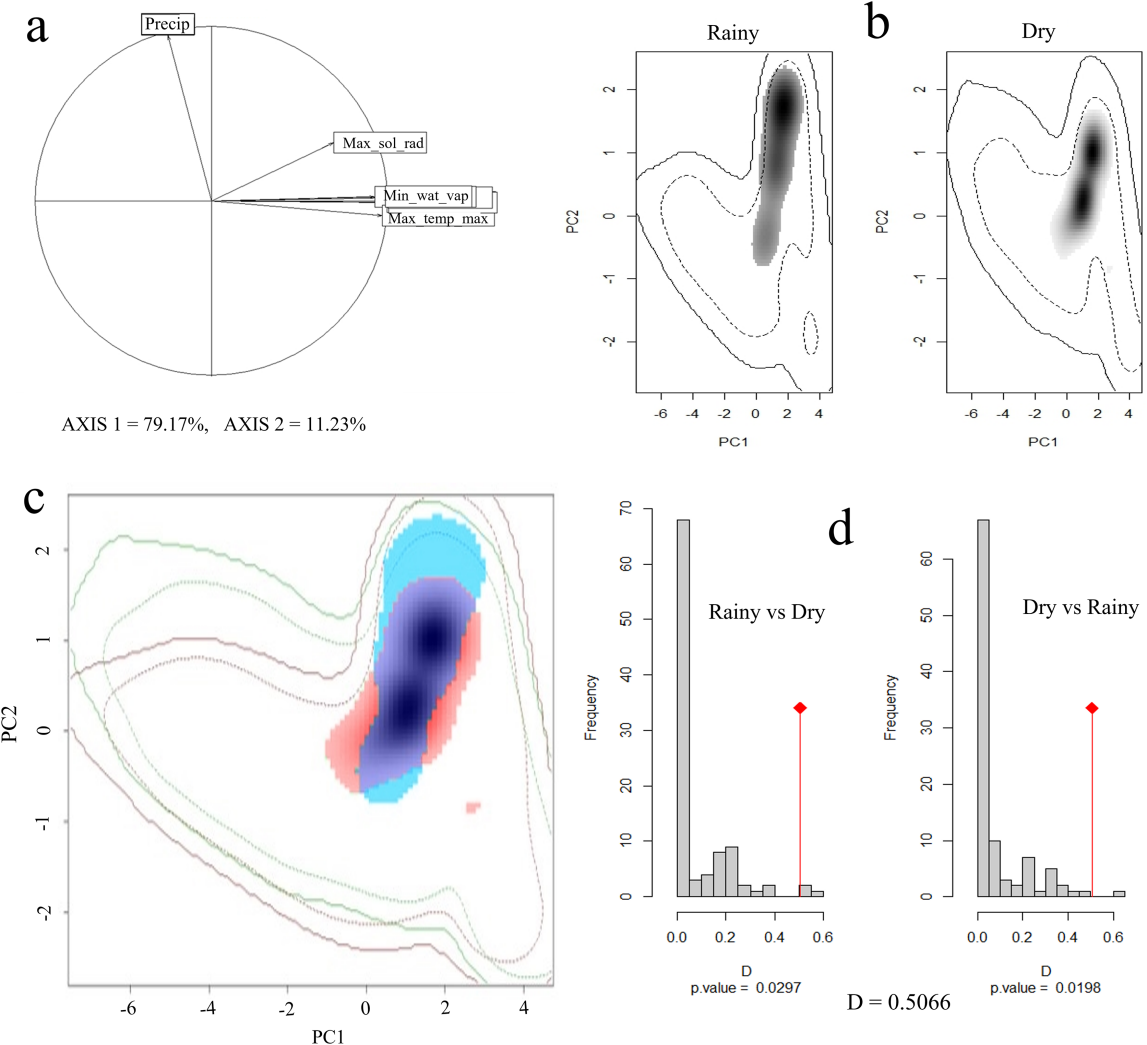
Seasonal climatic niche of the Short-crested Coquette hummingbird. (A) PCA with the climatic variables associated with each axis, (B) individual climatic niches in the rainy and dry seasons, (C) climatic niche overlap between the dry and the rainy seasons, (D) histogram from the niche similarity test between them. The shades of gray (in (B)) show the density of occurrences, while in (C), the red color indicates the climatic niche in the dry season, and blue color the niche in the rainy season.

Principal component analysis (PCA) indicated that 90% of the total variation is explained by the first two axes, with 79% and 11%, respectively. The variables that contributed the most to PC1 were maximum solar radiation, maximum temperature, minimum temperature, and water vapor pressure, while for PC2 it was precipitation (Fig. 4A).

In the rainy season, the SCCH used a broader range of climatic conditions than in the dry season (Fig. 4B). The Schoener’s D metric for the niche similarity test between the two seasons was D = 0.50 (*P* < 0.05; Fig. 4C), indicating that the observed overlap value is moderate but significant in relation to what would be expected by chance.

## Discussion

Our results revealed a high overlap in the potential distribution of the SCCH during the dry and rainy seasons, although in the dry season, the distribution reached its lowest limit and during the rainy season, its highest limit of the elevational gradient used by this species. These expansions in the elevational distribution are related to food availability (both flowers and fruits). Thus, changes in the elevational distribution of the species, are directly driven by the phenology of the flowers and fruits consumed by this hummingbird, although indirectly driven by climate. Hence, the climatic niche turned out to be moderately similar between both seasons, even though some climatic variables such as precipitation, water vapor pressure, and minimum temperature, are significantly different between seasons.

Our results also indicate that the total geographic range of the SCCH covers 671 pixels (or ~544 km^2^). Although other studies have suggested a larger potential distribution for this species (more than 2,700 km^2^, Sierra-Morales et al., 2016), these differences concerning our results could be directly related to the modelling methods, the approach (*i.e*., seasonally in this study) and the number of occurrence records used. Sierra-Morales et al. (2016) did not delimit a calibration area, which possibly allowed for greater overprediction. However, increases in occurrence records in areas a little distant from those typically recognized for this species in recent years (Sierra-Morales et al., 2019), suggest that its potential distribution is probably larger than historically recognized (70 to 90 km^2^, IUCN, 2024). On the other hand, the SCCH has a larger potential distribution area in the dry season compared to the rainy season. Specifically, this seasonal expansion is marked by a downward extension of 490 masl in the elevational gradient, extending the species’ range to lower altitudes. These findings confirm our hypothesis of an elevation-descent in the range of this hummingbird due to changes directly in food availability, but indirectly by changes in environmental conditions.

The expansion of the SCCH’s range toward lower elevations during the dry season coincides with the availability of flower resources at those elevations, as evidenced by our results. In the study area, the dry season promotes flowering peaks, as observed throughout the Neotropics (Wolf, 1970; López-Flores et al., 2024). This increase in the density of flowering plants occurs mainly during the months with greater solar radiation at intermediate to low elevations, enabling the coexistence of the SCCH with at least 16 other hummingbird species (Navarro, 1992; Almazán-Núñez et al., 2020, 2024). These changes in food availability would condition the elevational movement of the SCCH between both seasons, particularly due to greater flower production at higher temperatures (Pau et al., 2013), as well as the production of fruits on which this hummingbird feeds at lower temperatures (Morales-Martínez et al., 2024).

Some hummingbird species, particularly those with restricted distributions, tend to occupy habitats with less pronounced climatic seasonality (Rahbek & Graves, 2000). In our study, two of the five climate variables (maximum temperature and solar radiation) used by the SCCH did not show significant changes. However, in other studies, particularly in long-distance migratory hummingbirds or even in resident species that inhabit ecosystems with high seasonality, such as tropical dry forests, the variations in climatic conditions are significantly different for most variables (Hernández-Hernández et al., 2022). When unfavorable environmental conditions occur during certain periods, hummingbird species, particularly widely distributed ones, such as the Mexican Violetear, Amethyst-throated Mountain-gem, and Garnet-throated hummingbird, undertake local or altitudinal migrations in search of food resources (Martínez-Roldán, Pérez-Crespo & Lara, 2024). However, in microendemic species, these movements appear less evident. For the SCCH, observations have shown local movements to lower elevations during the dry season, possibly driven by the decline in available resources in those areas under colder conditions at higher elevations from November to January. Conversely, lower elevation sites experience an increase in resource availability from March to April, prompting this species to shift its distribution to exploit these seasonal changes, aligning with the findings of Arizmendi et al. (2021). This highlights the hummingbird’s ability to use seasonally varying environmental conditions and the crucial role that food availability plays in shaping its movements.

As demonstrated in our study, the SCCH performs movements in concert with flowering or fruiting, increasing its geographic distribution at higher elevations during the rainy season (May–July). Thus, as other hummingbirds, the SCCH require a constant food supply due to the high metabolic rates required by homeothermic bodies (Abrahamczyk et al., 2011; Figs. 1C, 1D), which in turn demand intensive foraging that enables them to visit flowers during rainy weather when pollinating insects are unable to fly (Lawson & Rands, 2019). This highlights the potential importance of the SCCH’s in pollination interactions, as has been described in other microendemic hummingbirds (*i.e*., *Eriocnemis mirabilis* in Colombia; Ramírez-Burbano et al., 2017). These seasonal changes in their distribution also require inter- or intraspecific interactions with other hummingbird species to allow their maintenance (López-Segoviano et al., 2023), as occasionally, these relationships do not favor the presence of other species, leading to competition and territorial displacement. We observed that several hummingbird endemic species to the Sierra Madre del Sur (SMS), such as White-tailed hummingbird, and widespread species such as Berylline hummingbird and Sparkling-tailed hummingbird, exhibit territorial behavior and competition for resources with the SCCH. However, in our study area, interactions between hummingbirds are largely influenced by tree stratification, allowing for coexistence despite their exploitation of similar resources (López-Flores et al., 2024). Furthermore, there could be a time split in the foraging of SCCH with other larger or dominant hummingbirds, such as Rivoli’s hummingbird and Long-billed Starthroat, as in trees with high flower density, it was not common to observe this species in the early hours of the morning. This phenomenon, known as diurnal niche partitioning, is a common pattern observed in hummingbird species in other regions of Mexico (Lara, Lumbreras & González, 2009).

Our results also indicated that, although the SCCH undergoes temporal changes in its distribution, its climatic niche shows a moderate overlap throughout the year (Rödder & Engler, 2011). This moderate overlap can be explained by two main factors. First, microendemic species typically exhibit narrow physiological tolerance ranges (Platania & Gómez-Zurita, 2023), and therefore, their ecological and environmental requirements tend to be more specific (Arizmendi et al., 2021; Rojas-Soto et al., 2021; Barreto et al., 2023). Consequently, under Hutchinson’s niche definition (Hutchinson, 1957), these species might possess a narrow fundamental niche according to their physiological capacities, and a narrow realized niche limited by the availability of resources in their distribution areas (Suarez & Gass, 2002).

There are no studies on niche similarity between seasons in microendemic species; and those that exist have been conducted mostly on species with broader distributions, such as the Broad-tailed hummingbird and Baird’s sparrow, both long-distance migratory species where the climate between breeding and winter seasons clearly defines two environmental usages in its niche (Peña-Peniche, Ruvalcaba-Ortega & Rojas-Soto, 2018; Hernández-Hernández et al., 2022). This pattern of shift in climatic niche usage, commonly observed in widely distributed species, would not be expected to be as pronounced in a microendemic species (Hu et al., 2016) as in the SCCH. However, despite its restricted geographic distribution (Velasco, Martinez-Meyer & Flores-Villela, 2018), the species’ needs for food supply between seasons probably allowed it the ability to use climatic environments moderately different in a short-distance movements.

Another factor that may explain the moderate overlap in the seasonal niche concerns the predominantly humid conditions in the occupied area, which includes humid mountain forests, such as cloud and tropical semideciduous forests (Almazán-Núñez et al., 2020). These ecosystems provide dense cover due to the humid conditions prevailing for most of the year (Luna-Vega, Espinosa & Contreras-Medina, 2016), thereby promoting the requirements for the presence of this species.

The Sierra Madre del Sur is one of the regions with the highest humidity levels in Mexico (Hernández-Cerda, Azpra-Romero & Aguilar-Zamora, 2016), retaining moisture from the Mexican Pacific Slope (Trasviña & Barton, 2008) and largely driven by the complex orography of the region (Velasco-de León et al., 2016). The isolated mountain range to which the SCCH is restricted within the Sierra Madre del Sur and particular climatic conditions in which the species is found (Almazán-Núñez et al., 2024), may be associated with the fragmented distribution patterns in the so-called “sky islands” (McCormack, Bowen & Smith, 2008). This pattern corresponds to montane habitats geographically subdivided and isolated among different mountain ranges, which have driven the presence of taxa with restricted distributions and population differentiation in response to local geographic and climatic conditions (Rocha-Méndez et al., 2024). This suggests that the SCCH has acquired its own ecological identity about other species of coquettes distributed in southern Mexico, Central, and South America, as evidenced even for lineages that inhabit the same geographic region across different latitudes (such as *E. ridgwayi, E. poliocerca,* and *E. cyanophrys* in the SMS, Rocha-Méndez et al., 2024). However, further studies could evaluate this hypothesis of ecological niche differentiation among species of the coquette complex.

Species’ physiological constraints and physical barriers might play a crucial role as promoters in the seasonal changes (Toro-Cardona, Parra & Rojas-Soto, 2023). For the SCCH, the climatic niches between the dry and rainy seasons overlap by 50%. However, although the humidity range is greater in the rainy season due to increased precipitation, changes in the climatic conditions used by this species are not markedly abrupt, compared to the dry season. This suggests that the SCCH uses moderately similar climatic conditions between seasons where variations comes from precipitation mainly, although maximum temperature and insolation are relatively stable throughout the year (Vidal-Zepeda, 1990).

Accordingly, this hummingbird changes its geographic distribution following food availability along the elevational gradient between the two seasons, which could define its realized niche (*i.e*., Eltonian niche). Some studies suggest that long-distance migratory species are more likely to encounter wide climatic variations in their reproductive and wintering areas, leading to differentiation in their seasonal climatic niche (Dufour et al., 2020; Ponti et al., 2020; Hernández-Hernández et al., 2022). These climatic variations, in which humidity essentially drives the dynamics of biotic communities, tend to be less frequent in species with local distributions, as is the case with the SCCH. It has been suggested that some factors driving species distributions at local scales are primarily related to habitat availability, microclimates, and phenological events in plant species, including flowering and fruit formation (Lembrechts, Nijs & Lenoir, 2019; Zurell & Zimmermann, 2024), from which the SCCH is fed. Although hummingbirds are primarily nectar-feeders and pollinators, hummingbird frugivory is a widespread phenomenon across the Americas (Missagia et al., 2025), but not documented for SCCH; hence, our observation is the first record, also suggesting that they may be using fruit as an additional energy source.

## Conclusions

For microendemic and critically endangered species on a global scale, like the SCCH, understanding the factors limiting geographic distributions and climatic niches is crucial from a conservation perspective. Seasonal changes in the distributional area of the SCCH enable us to visualize the potential limits of physiological tolerance in contrasting environmental conditions (dry and rainy seasons). Given the present and future impacts of climate and land use changes on the reduced distribution of this species, understanding its adaptive response to environmental changes and food availability can inform conservation decisions for this species and its habitats. This is particularly relevant for the SCCH, as it maintains moderately similar climatic requirements throughout the year, demonstrating a certain physiological constraint that, besides to its small population size, would condition the long-term survival of this species.

Our results suggest that climate does not directly influence the geographical and ecological distribution of the SCCH between the dry and rainy seasons. Rather, the observed distributional changes are more likely linked to the phenology of its food resources—flowers in the dry season and fruits in the rainy season—both of which are indirectly influenced by climate. This species’ monitoring of food availability within its range could also influence the moderate overlap between its climatic niche in both seasons. However, given the crucial role played by the plants used as food resources, the potential effects of climate change on these plant species needs to be further evaluated. This is particularly relevant because flower production trends are changing due to rising temperatures under climate change scenarios (Correa-Lima et al., 2019). This will provide a more comprehensive framework for informing the most viable conservation scenarios for this high-priority species on a global scale.

## Supplemental Information

10.7717/peerj.20312/supp-1 Supplemental Information 1 Short-crested coquette hummingbird occurrence data for the dry and rainy seasons used to generate species distribution models and climatic niche models. With these data, both potential distribution models and climatic niche models were performed.

10.7717/peerj.20312/supp-2 Supplemental Information 2 R codes used for species distribution and climate niche models for SCH.

10.7717/peerj.20312/supp-3 Supplemental Information 3 Variables obtained from WorldClim v. 2.1. The variables are shown for the rainy and dry seasons. The 12 files (.Asc) correspond to one for each month of the year.

10.7717/peerj.20312/supp-4 Supplemental Information 4 Relative contributions of environmental variables to the SCCH distribution model in Maxent during the rainy and dry seasons.

10.7717/peerj.20312/supp-5 Supplemental Information 5 List of flowering and/or ripe fruit plant species observed along the transects and consumed by the SCCH along the elevation gradient. The plant species consumed by the SCCH were obtained from *Arizmendi et al*. (*2021*), *López-Flores et al*. (*2024*), *Bravo-Galindo et al*. (*2025*), and *personal observations*.

## Additional Information and Declarations

### Data Availability

The following information was supplied regarding data availability:

The occurrence data of the Short-crested Coquette Hummingbird are available in the Supplemental Files.

Raw data are available at GBIF:

- https://www.gbif.org/es/occurrence/search?taxon_key=2477012&occurrence_status=present.

The Short-crested Coquette is available at eBird: https://ebird.org/species/shccoq.
