# Supplementary material for: Geographical and seasonal distribution of the Short-crested Coquette hummingbird: a microendemic and endangered species
Source: PeerJ. 2025 Nov 11;13:e20312. doi: 10.7717/peerj.20312 (PMC12617369; doi:10.7717/peerj.20312)
Supplement: Supplemental Information 3 — The variables are shown for the rainy and dry seasons. The 12 files (.Asc) correspond to one for each month of the year. [file peerj-13-20312-s003.docx]

**Supplementary Information**

**Table S1** Variables obtained from WorldClim v. 2.1. The variables are shown for the rainy and dry seasons. The 12 files (.Asc) correspond to one for each month of the year.

| **Variables** | **Bios (Asc)** | **Months and Asc ID for rain season** | **Months and Asc ID for dry season** |
| --- | --- | --- | --- |
| Minimum temperature (°C) * | 12 | May (5), June (6), July (7), August (8), September (9), October (10) | November (11), December (12), January (1), February (2), March (3), April (4) |
| Maximum temperature (°C) * | 12 | May (5), June (6), July (7), August (8), September (9), October (10) | November (11), December (12), January (1), February (2), March (3), April (4) |
| Average temperature (°C) | 12 | May (5), June (6), July (7), August (8), September (9) October (10) | November (11), December (12), January (1), February (2), March (3), April (4) |
| Precipitation (mm) * | 12 | May (5), June (6), July (7), August (8), September (9), October (10) | November (11), December (12), January (1), February (2), March (3), April (4) |
| Solar radiation (Kj m^-2^ day^-1^) * | 12 | May (5), June (6), July (7), August (8), September (9), October (10) | November (11), December (12), January (1), February (2), March (3), April (4) |
| Wind speed (ms^-1^) | 12 | May (5), June (6), July (7), August (8), September (9), October (10) | November (11), December (12), January (1), February (2), March (3), April (4) |
| Water vapor pressure (kPa) * | 12 | May (5), June (6), July (7), August (8), September (9), October (10) | November (11), December (12), January (1), February (2), March (3), April (4) |

Variables used in the climatic niche analysis are indicated with an asterisk.
