# Supplementary material for: Geographical and seasonal distribution of the Short-crested Coquette hummingbird: a microendemic and endangered species
Source: PeerJ. 2025 Nov 11;13:e20312. doi: 10.7717/peerj.20312 (PMC12617369; doi:10.7717/peerj.20312)
Supplement: Supplemental Information 4 [file peerj-13-20312-s004.docx]

**Supplementary Information**

**Table S2** Relative contributions of environmental variables to the SCCH distribution model in Maxent during the rainy and dry seasons.

| **Rainy season** | | |
| --- | --- | --- |
| **Variable** | **Percent contribution** | **Permutation importance** |
| Radsol_min | 37.2 | 34.6 |
| Precip | 21.5 | 3.9 |
| Tempmin_min | 13.4 | 13.4 |
| Tempmax_min | 10.5 | 34.4 |
| Radsol_max | 9.2 | 0 |
| Tempmax_max | 5.1 | 13.7 |
| Vagua_min | 3.1 | 0 |
| Vagua_max | 0 | 0 |
| Tempmin_max | 0 | 0 |
| **Dry season** | | |
| Radsol_max | 53.1 | 4.1 |
| Tempmax_min | 20.7 | 45.8 |
| Radsol_min | 7.9 | 0.2 |
| Tempmax_max | 7.6 | 18.9 |
| Tempmin_min | 6.9 | 14.3 |
| Vagua_min | 2.7 | 9.6 |
| Precip | 1.2 | 7.1 |
| Tempmin_max | 0 | 0 |
| Vagua_max | 0 | 0 |
