# Supplementary material for: Geographical and seasonal distribution of the Short-crested Coquette hummingbird: a microendemic and endangered species
Source: PeerJ. 2025 Nov 11;13:e20312. doi: 10.7717/peerj.20312 (PMC12617369; doi:10.7717/peerj.20312)
Supplement: Supplemental Information 5 — The plant species consumed by the SCCH were obtained from Arizmendi et al. (2021), López-Flores et al. (2024), Bravo-Galindo et al. (2025), and personal observations. [file peerj-13-20312-s005.docx]

**Supplementary information**

Table S3 List of flowering and/or ripe fruit plant species observed along the transects and consumed by the SCCH along the elevation gradient. The plant species consumed by the SCCH were obtained from *Arizmendi et al.* (*2021*), *López-Flores et al.* (*2024*), *Bravo-Galindo et al.* (*2025*), and *personal observations*.

| **Plant species** | **Geographic coordinates** | **Locality** | **Elevation** |
| --- | --- | --- | --- |
| *Clethra fragans* | 100°19'20.64"O; 17°13'35.75"N | Santiago Unión | 700 |
| *Inga vera* | 100°19'20.64"O; 17°13'35.75"N | Santiago Unión | 700 |
| *Conostegia xalapensis* | 100°19'20.64"O; 17°13'35.75"N | Santiago Unión | 700 |
| *Inga vera* | 100°20'25.97"O; 17°13'30.73"N | Santiago Unión | 700 |
|  |  |  |  |
| *Inga vera* | 100°18'26.72"O; 17°15'42.88"N | Rio Santiago | 900 |
| *Sommera grandis* | 100°18'26.72"O; 17°15'42.88"N | Rio Santiago | 900 |
| *Coffea arabica* | 100°18'26.72"O; 17°15'42.88"N | Rio Santiago | 900 |
| *Clethra fragans* | 100°18'26.72"O; 17°15'42.88"N | Rio Santiago | 900 |
|  |  |  |  |
| *Clethra fragans* | 100°14'47.97"O; 17°17'13.61"N | San Vicente de Jesús | 1100 |
| *Conostegia xalapensis* | 100°14'47.97"O; 17°17'13.61"N | San Vicente de Jesús | 1100 |
| *Inga vera* | 100°14'47.97"O; 17°17'13.61"N | San Vicente de Jesús | 1100 |
| *Sommera grandis* | 100°14'47.97"O; 17°17'13.61"N | San Vicente de Jesús | 1100 |
| *Vernonanthura cordata* | 100°14'47.97"O; 17°17'13.61"N | San Vicente de Jesús | 1100 |
| *Salvia* spp. | 100°14'47.97"O; 17°17'13.61"N | San Vicente de Jesús | 1100 |
| *Clusia salvinii* | 100°14'47.97"O; 17°17'13.61"N | San Vicente de Jesús | 1100 |
|  |  |  |  |
| *Conostegia xalapensis* | 100°11'30.57"O; 17°19'29.36"N | El Paraiso | 1300 |
| *Inga vera* | 100°11'30.57"O; 17°19'29.36"N | El Paraiso | 1300 |
| *Vernonanthura cordata* | 100°11'30.57"O; 17°19'29.36"N | El Paraiso | 1300 |
| *Clethra fragans* | 100°12'24.14"O; 17°19'16.43"N | El Paraiso | 1300 |
| *Clusia salvinii* | 100°12'24.14"O; 17°19'16.43"N | El Paraiso | 1300 |
| *Sommera grandis* | 100°12'24.14"O; 17°19'16.43"N | El Paraiso | 1300 |
| *Coffea arabica* | 100°12'24.14"O; 17°19'16.43"N | El Paraiso | 1300 |
|  |  |  |  |
| *Sommera grandis* | 100° 8'38.13"O; 17°20'47.01"N | La Pintada | 1500 |
| *Vernonanthura cordata* | 100° 8'38.13"O; 17°20'47.01"N | La Pintada | 1500 |
| *Clethra fragans* | 100° 8'38.13"O; 17°20'47.01"N | La Pintada | 1500 |
| *Inga vera* | 100° 9'28.22"O; 17°20'10.88"N | La Pintada | 1500 |
| *Conostegia xalapensis* | 100° 9'28.22"O; 17°20'10.88"N | La Pintada | 1500 |
| *Inga vera* | 100° 9'28.22"O; 17°20'10.88"N | La Pintada | 1500 |
| *Sommera grandis* | 100° 9'28.22"O; 17°20'10.88"N | La Pintada | 1500 |
| *Clethra fragans* | 100° 9'28.22"O; 17°20'10.88"N | La Pintada | 1500 |
|  |  |  |  |
| *Clethra fragans* | 100°10'40.29"O; 17°25'14.29"N | Nueva Delhi | 1700 |
| *Inga vera* | 100°10'40.29"O; 17°25'14.29"N | Nueva Delhi | 1700 |
| *Vernonanthura cordata* | 100°10'40.29"O; 17°25'14.29"N | Nueva Delhi | 1700 |
| *Sommera grandis* | 100°10'40.29"O; 17°25'14.29"N | Nueva Delhi | 1700 |
| *Conostegia xalapensis* | 100°10'40.29"O; 17°25'14.29"N | Nueva Delhi | 1700 |
